# American Dental Association

**Published:** 1896-12

**Authors:** 


### Reports of Society Meetings.

### AMERICAN DENTAL ASSOCIATION.

(Continued from page 737.)

*August 5, 1896.—Second Day.—Morning Session.*

Meeting called to order at 9.15 by the President, Dr. Crawford. The minutes of the previous session were read and approved.

The Executive Committee reported that they have appropriated funds for the payment of the expenses of the Committee on the National Museum and Library at Washington.

Section V., Materia Medica and Therapeutics, presented a paper by Dr. Joseph W. Wassall, of Chicago, entitled "The Disinfection of Pulpless Teeth." The same section also exhibited some specimens prepared by Dr. King, of Fremont, Nebraska.

Dr. Wassall's paper follows:

"Are current methods of practice in the treatment of teeth with necrotic pulps strictly in accordance with modern views of asepsis? Judged by what is said and written for societies, periodical literature, and text-books, which may be presumed to fairly represent the methods in common use, this question must be answered negatively. It would seem then that no apology is required in bringing before you so trite a subject; on the contrary, if the inference which my question raises is correct, it is eminently proper that this representative body should give the matter its serious consideration.

"Given a tooth with a decomposing pulp, what is the nature of the pathological state with which we have to deal? We find a condition in which the pulp-chamber, root-canals, and dentinal tubules are loaded with putrefactive animal matter. One has but to recall the large amount of soft tissue contained in dentine and its formative organ to realize the danger of the situation. The recent analyses of dentine made by Dr. G. V. Black and reported by him in the *Dental Cosmos* for May, 1895, gives an average of 25.36 per cent. of organic matter, and 10.06 per cent. of water. The pulp would constitute additional soft tissue amounting to about one-third of the bulk of the dentine.

"Now, while the removal of this necrotic mass from the chamber and canal, and the disinfection and filling of the vacated space is a manifest necessity, and is the general teaching and practice, I contend that there is not a general appreciation of the fact that the dentine itself continues to remain aseptic. It is for the purpose of emphasizing the necessity for scientifically sterilizing this putrid zone of dentine that this paper is offered.

"What is the chemical and physical nature of this putrescent soft tissue? Putrefaction is a fermentative decomposition in dead animal tissue, produced by a class of micro-organisms termed saprogenic bacteria. Professor W. D. Miller, in his 'Micro-organisms of the Human Mouth, 1890,' says, 'The fermentation of nitrogenous, and more particularly of albuminous substances, which is accompanied by the development of large quantities of gaseous and stinking products, is called putrefactive fermentation, or simply putrefaction.' The definitions given in the latest editions of Foster, Gould, and Dunglison are almost identical with the above.

"McFarland ('Pathogenic Bacteria,' 1896) describes the chemical changes taking place in the process of putrefaction as follows: 'The first step seems to be the transformation of the albumins into peptones, then the splitting up of the peptones into a large number of gases, acids, bases, and salts. In the process the innocuous albumins are frequently changed to tox-albumins, and sometimes to distinct animal alkaloids known as ptomaines.'

"To quote Miller again (*ibid.*): 'This name (ptomaines) has been given to certain waste products of bacteria closely resembling vegetable alkaloids, which are formed in putrefying mixtures.'

"Ptomaines have exceedingly poisonous properties. According to Brieger (an authority on this subject), they may have an action on the animal body similar to snake poison and curare. Extended researches into the nature of these substances made by Bergman, Panum, Brieger, Suelzer and Sonnenschein, Vaughan, and others prove that their toxic properties abundantly suffice to produce the violent inflammatory phenomena which are observed to follow the contact of putrescent pulp matter with the living tissue. Is it not apparent, then, that the mere enunciation of the canal contents and a single application of any known antiseptic is insufficient to sterilize or render permanently innocuous these deleterious alkaloids? Yet every day such doctrine is enunciated, and such methods are advocated and have authority because they pass unchallenged.

"There are three classes of cases in which we have pollution of the dentine by putrefactive products:

"1. Teeth, the pulps of which have perished from encroachment of caries, the pulp-chamber being open.

"2. Teeth, the pulps of which have died from proximity of a large filling, attempts at capping, or insufficient sterilization of the layer of caries allowed to remain over a pulp.

"3. Pulpless teeth, the canals of which have been imperfectly filled or sterilized, or both.

"A tooth, the pulp of which has been devitalized intentionally, is excluded from consideration in this connection, for the reason that under ordinary aseptic precautions putrefaction does not occur.

"The essential pathological condition to be recognized is the same in all three cases given. We have dead dentine infiltrated with matter highly irritating and poisonous to living tissue in intimate contact with vital cementum, which in turn is closely enveloped in pericementum.

"What must the effect be on the cementum and peridental membrane of the *materies morbi* which are present in the underlying dentine? There is no escape from the conclusion that it must account for many morbid conditions, the etiology of which is sometimes obscure. There are no doubt exceptions (many of you may call them to mind) of teeth in this state which cause no discomfort. A little more time may prove that even these cases will not remain quiescent. Unquestionably this condition is responsible for numerous affections more or less difficult of diagnosis, varying from reflex neurosis to remotely situated abscesses, the only subjective symptoms in the causative tooth discoverable being a slight sense of lameness in mastication or to palpation.

"What treatment does this pathological state indicate? It will not be uninteresting to first notice some of the methods which have of late been prominently set forth.

"The transactions of the World's Columbian Dental Congress contain an article by Professor Miller, entitled 'Concerning Various Methods advocated for obviating the Necessity of extracting Devitalized Tooth-Pulps,' in which, while recognizing the greater value of complete root-canal filling, he advises for general practice the removal of the bulbous portion only of the pulp, and the covering over of the remaining portion with a tablet composed of slowly-dissolving antiseptics, such as sublimate and thymol combined. The distinguished author confesses that teeth so treated inevitably give trouble, if the victim lives long enough, which is condemnation sufficient. This, together with other variations upon the process of mummification, as advocated by Witzel, Herbst, and Soderburg, are only temporarily effective, because, to use Dr. Harlan's words, 'they will not stay mummified.' Is it not a fact that the only pulp which may be successfully mummified is the living pulp,—or, to be more accurate, the mummifying agent must be applied before pulp death. This would explain the observed efficacy of the oxychloride of zinc pulp-capping operation.

"Emil Schrier, at the same Congress, presented a method of destroying pulp-remains with a mixture of sodium and potassium, which is objectionable on the ground that the action of the drug does not extend into the entire depth of the tubules. Dr. J. Foster Flagg (whose name has far-reaching influence), in a paper read before the New Jersey State Dental Society (published in the *Dental Cosmos* for March, 1895), places the stamp of his approval upon this unscientific procedure. He says, 'I desire to combat to the utmost the claims of thorough removal of pulp-tissue as a matter of *first* importance.' He also makes use of a fifty-per-cent. solution of sulphuric acid for opening up the putrescent canals, which is contraindicated because it is a coagulator of albumen.

"Dr. M. L. Rhein, in a paper read before the Second District Dental Society of New York, and published in the *Dental Cosmos* for March, 1896, presents his method of treatment of acute and chronic alveolar abscess. The treatment he therein outlines is insufficient for the disinfection of putrid dentine. It is inadequate to the requirements of the condition, because the disinfectant applications are not continued for a long enough period of time. He also falls into the error of using coagulating agents for the first surgical proceeding, when it is quite probable that at the time the tubular structure of the dentine contains albuminous matter.

"Professor Abbott has written a work on dental pathology and practice, which, owing to the author's eminent position both as an educator and practitioner, will be widely read.

"It is particularly unfortunate for the younger members of the profession, whose methods are most likely to be influenced by it, that that chapter devoted to the consideration of this question should be so inconsistent with modern bacteriological knowledge. It is somewhat startling to find that 'until within a comparatively few years, there was no apparently rational or successful treatment for pulpless teeth except extraction.'

"Shades of Parmly, Harris, Townsend, Atkinson, and Maynard! The filling of root-canals has been orthodox dental practice for more than half a century. There are many gentlemen present at this meeting who have filled canals for thirty or forty years. Dr. McKellops told me yesterday that he recently saw a tooth still in the patient's mouth, in which he filled the root-canal with gold-foil over forty years ago. Dr. Corydon-Palmer has filled root-canals since 1839.

"Dr. Abbott imparts a lucid understanding of the fact of putrid dentine and the conditions to be overcome; but the treatment proposed is contrary to the commonest laws of asepsis. The triumph of modern surgery is secured by striving to prevent the entrance of germs into rather than their destruction after admission to a wound. How hazardous to teach that a pulpless tooth may be operated upon without first using the rubber dam! The neglect of this precaution against the ingress of the myriads of microorganisms, which are always present in the human mouth, is hardly excusable at this day. Dr. Abbott also recommends the use of bichloride solutions for syringing out the pulp *débris*. This is of doubtful utility, because, like nitrate of silver, it is at once precipitated in the presence of albumens, thus losing its germicidal and antiseptic powers. (McFarland.) It is also bad practice to pump zinc chloride through a tooth to cauterize a pus-sac until a course of treatment for sterilization of the dentine with diffusive disinfectants has first been completed.

"These few criticisms are submitted in order to substantiate the charge made at the opening of this paper, that much of the modern practice in the management of pulpless teeth does not conform to the present status of bacteriological science.

"The successful treatment of teeth contaminated with putrid pulp-matter would to my mind seem to depend upon the strict observance of two details of procedure,—

"1. The exclusive use of diffusible disinfectants.

"2. The repeated and continued application of the disinfectant dressings for a sufficient length of time.

"The reason why I give diffusible disinfectants the preference over coagulating drugs is because I am satisfied that their exhibition at the pulpal orifices of dentinal tubules forms a plug of coagulated matter which prevents the further ingress of the disinfecting agent, imprisoning within the tubules putrefactive matter which will be a permanent source of irritation to the cementum and pericementum. Again, if the position so ably maintained by Truman and Kirk is true (but which is not substantiated),—that the entire contents of the tubuli are coagulated,—there is nothing gained, for the resulting mass, it is well known, is suitable pabulum for micro-organisms. Hence, there would be no assurance that putrefaction would not recur within the tubules.

"As bearing upon the controversy regarding the relative merits of coagulating and diffusive disinfectants, I desire to draw attention to some recent experiments reported to the Iowa State Dental Society, in May, 1895, by Dr. Harlan. The starch and iodine test was employed. Freshly extracted teeth had their pulps removed and their foramina sealed. Root-canal dressings of coagulating disinfectants were then applied in the usual manner, and the teeth immersed in starch solution up to their anatomical necks. The characteristic blue color did not appear in the starchy solution, showing that the root had not been penetrated by the iodine. Iodine itself is a coagulator of albumen, so the experiment was also made by using the iodine alone in the same manner, omitting the carbolic acid. No indigo color appeared, demonstrating that its coagulating effect on the contents of the tubuli was an effectual bar to its diffusibility.

"It is difficult to reconcile the diametrically opposite results which seem to be obtained by Dr. Harlan on one side, and Drs. Truman and Kirk on the other. Perhaps the very complexity of the processes in experimentation, and the peculiar delicacy of the handling required, would account for some of the discrepancies. In the experiments, for instance, of Dr. Kirk, when the teeth were suspended in egg albumen, is it not possible that the apparent coagulum which is reported might not have been a growth of micro-organisms? The medium used and some of the conditions present would certainly be favorable to a culture. Then, again, a very important point seems to have been entirely overlooked. If the putrefaction of albumen has advanced far enough we no longer have albumen but ptomaines, which are not coagulable. Thus, it would be an easy matter to demonstrate either side of the question tenable according to the degree of decomposition of the animal matter within the dentine. This latter point seems to force us to the conclusion that, inasmuch as it is impossible with our present knowledge to accurately determine the stage of putrefaction with which we have to deal in a given case, the only safe course is to assume that coagulable material is present. Albuminous peptones and ptomaines are soluble in the diffusible penetrating drugs used in this condition, but are not soluble in coagulating agents unless such agents are diluted to a degree where they are no longer efficacious as disinfectants. In the investigations thus far published, the preponderance of evidence seems to be in favor of the avoidance of the coagulating drugs in the roots of pulpless teeth. The second requisite to success in the disinfection of putrid dentine mentioned was the element of time. How long should the dressings be kept in the canal before it is proper to fill the canal? Until the dentine is permeated throughout its entire depth and until all micro-organisms and their spores may reasonably be expected to be destroyed.

"My own observation is that these results are obtained in not less than twelve days, oftener sixteen or twenty, and in some few cases a longer course of treatment is required.

"The dressings should be changed every four days until no stain or odor is perceived on the cotton dressing other than the drug employed. Even though the dressing may come away clean on the fourth or eighth day, and you are morally certain that the bacteria are killed, it is imperative that the use of the drugs be continued for the full period in order to kill any spores which may be present, for they afford much greater resistance to germicides, and hence require more time for their destruction."

DISCUSSION.

*Dr. Abbott.*—I have been particularly interested in Dr. Wassall's paper for more reasons than one. In the first place, I am naturally inclined to defend my own position as taken in my little book that was published within the last year. I am inclined also, as a teacher, to want always the very best thing I can get in the way of treatment of diseased teeth of this kind. Let us see what scientific treatment really consists of. As I understand it, the crowning point of all scientific treatment is the result. If the result is favorable, we want nothing better as far as treatment is concerned. You may say there may be germs and spores left here and there, but suppose they are put in a condition in which they are unable to accomplish anything? We have teeth that have been going-on for twenty-five or thirty years, and with the modern treatment for ten or twelve years without any disturbance whatever. Do we want anything better? We have had several stages of this scientific treatment. We had a man here not many years ago who would get up and state that the only scientific way to treat a pulpless tooth was to take a drill and go right through it, and if you could not get through that way, go through the side or anywhere, as long as the patient felt it.

Another method that was presented is the one spoken of by Dr. Wassall, that the tooth should be treated day after day. A tooth is opened, cleansed out with antiseptics, plugged up with an antiseptic, if you please, and is supposed to be cleansed of all putrefactive material at that time. You put in your dressing and leave it there for three or four days or more, and then take it out, and a fresh batch of bacteria that are always floating about are allowed to get in. It is cleansed out again, and again filled up with some material. This is done eight or ten times, and every time it is opened you allow a fresh batch of germs to get in and bring back the conditions you had when you began. In many of these cases I have taken out the material supposed to be an antiseptic plug, and had pus and blood flow out. The next point is the treatment which has been condemned in the paper, and which I practised for ten or twelve years. I have yet to see one single failure in the treatment. The moment the tooth is opened, it is put under the influence of a powerful antiseptic and kept that way from that time on, and nothing allowed to get in the canal except the material I put in as an antiseptic, and before the patient leaves the chair I fill the tooth instead of drying it out, spending half an hour or an hour with a very uncertain procedure in drying; in fact, it is one of those things that many of the older men will agree with, when I say, it is almost impossible to dry a canal. We have tried to dry it with hot air to get all the moisture out of the canaliculi and sterilize the canaliculi. Is it accomplished? Is there anything in that practice that commends itself to a man who is constantly at work over the mouth and sees the results of operations? The very thing that is attempted to be accomplished is not accomplished. If the canaliculi were made entirely dry, the tooth would be likely to break and the enamel would probably break from the contraction. My method of treatment is one that any one can read, because I have published it in as careful a manner as I knew how. I have said nothing that practice has not fulfilled to the letter.

All teeth in which the pulps are dead, are treated and filled at once. I consider a coagulating material one of the best things to use. I consider it important that all the material in the canaliculi should be plugged up; stop all change if we can do it. I have treated abscesses in the manner in which I do now for fifteen years. Fifteen years ago a gentleman in our State society said that there was no material in the whole Pharmacopœia as valuable in the dentist's hands as chloride of zinc, and I believe he was right. No material will accomplish the work as thoroughly as that does. I use it in the treatment of alveolar abscesses, and I fill them at once, and the failures are so slight that they are hardly worth mentioning. You will not fail once in a hundred times. If that is not scientific treatment I have nothing further to say.

*Dr. Patterson.*—It does seem to me that any one who has seen a tooth, which has been in a diseased condition for some time, filled in the manner which Dr. Abbott describes, without any care to disinfect the zone of dentine, and afterwards examined that tooth when it is extracted, or if it still remains in the mouth (we often have to do that, entering into the dentine which has not been sterilized, and observing the condition in which we find it), ought to be satisfied that Dr. Abbott's method is an incorrect one,—that there must be time and the proper diffusible medicine to penetrate to all parts of that dentine. If it is not done we find it in a deplorable condition. The odor will reach your nostrils several feet away, whereas, if you treat the dentine with modern methods and afterwards enter the zone of dentine, you will not find it in the same deplorable condition as in the other case. That practical way of finding out the condition of the dentinal zone, after it has been treated one way and then the other, would certainly satisfy me that the method of not paying any particular attention to sterilizing that zone of dentine is the incorrect method, and must ever remain the incorrect method. Many of the low-grade troubles which we find around the teeth in after years must, in my opinion, result from leaving the zone of dentine in that condition. I cannot conceive how it can be otherwise; and when we can sterilize it according to modern methods, according to the method described by Dr. Wassall, why should we take any risk? It appeals to my common sense, at least, and I do not propose to take any risk, because my experience has proved that it is a risk. My examination of the teeth which have not been treated by the modern method has convinced me that they are in a condition which will absolutely, in nine cases out of ten, cause some pericemental trouble.

*Dr. Morgan.*—I want to correct some assumptions with regard to the present treatment being a modern one. When I was a student, in 1847, I saw teeth treated by Professor Harris very much the same as they are to-day, except that he followed the method of immediate root-filling. The main difference between the treatment then and now, in the beginning, was that he drilled out and opened the root-canals as thoroughly as he was able to do; then he used an antiseptic treatment, but followed it immediately with his filling. If you examine the old *American Journal of Dental Science*, about 1854 or 1855, you will find a series of articles by Dr. Ballard, of London, in which the basal treatment that is followed now is laid down distinctly and clearly. He opened the root-canal as far as he could, which was not done with the intention of removing so much of the soft portions as he did, but it was a benefit because it was out of the way; then he treated it with antiseptics exactly upon the principle which you do now. He used the antiseptics then, and took a thread of floss silk, immersing it in the medicament, and hermetically sealed it in the cavity. When he took out his filling he took hold of the silk, and his test was the test of odor. He would treat the tooth for ten or fifteen days, and then put in a gold filling. If I had a few patients here from Nashville, I could show you some front teeth, in at least two mouths, that were filled in the early fifties, where the teeth are intact to-day and are doing good service. One of the most important matters in connection with the treatment is the differentiation as to the abnormal or normal tendencies of patients. In patients of good constitutions and bilious temperaments the teeth are retained much longer, but in scrofulous patients you have very much more trouble in getting your teeth in a proper condition to fill, and you will have results that are much less endurable. Take the colored people of the South; you can scarcely preserve a pulpless tooth in their mouths. Take a mulatto, and you can hardly save it at all. They take on inflammatory conditions much easier, and the result is much more grave because of the loose structure of the tooth. I have been practising dentistry over fifty years, and in all that time I have seen two mulattoes, twenty-one years old, who had sound sets of teeth. Such is the predisposition to disease where there is mixed blood; such is the readiness with which they run into putrefaction that their teeth are destroyed in early life. You will find that, in our race, the teeth of those who are inclined to scrofulous diseases in any form are much softer and require more care and a closer differentiation between those that may be preserved and those that cannot. I ask those of you who use this antiseptic treatment, and keep it there for days and weeks, to look up the old journals and see Dr. Ballard's treatment. We are not using the antiseptics now that we used then, and we have made much progress in that direction and our field is being widened, but I assure you that in the main that which Dr. Patterson called "modern treatment" is not modern in the sense that it has occurred within a few years.

*Dr. Patterson.*—The only thing I advocated was not that antiseptics were not used, but the diffusible antiseptics which we use now instead of the escharotics and the coagulants. That was what I referred to by the modern method.

*Dr. Abbott.*—Dr. Patterson says if you open one of these teeth you get a disagreeable odor. There is all the difference in the world in the manner in which teeth are treated. I suppose I have treated more teeth of this kind than any man in America, and I have yet to see one that has been properly filled with oxychloride of zinc, in which there is any odor from the canal in one month, one year, or six years.

*Dr. Barrett.*—There is an old aphorism which, it seems to me, is perhaps the most applicable, especially in dental practice, of anything with which we are acquainted,—"The middle course is the best." I believe that to be true in the subject which is now under consideration. The question was mainly in relation to the septic conditions of dentine, not of pulp-canals, not of the decomposition of pulp-tissue, but of the dentine and the contents of the dentinal tubuli. They are of an albuminoid character. They are unorganized tissue. The fact that the remedy which I propose to use belongs to or does not belong to the class of coagulants is not that which determines my use of it. It is not that which causes me either to use or reject it, because the albuminoid contents of the dentinal tubuli will coagulate whether you do or do not use a distinct coagulating remedy. They will coagulate spontaneously if you supply the opportunity and give them the conditions to which they will be exposed. It is the primary step in the breaking down of the dentinal tubuli of their protoplasmic contents; and when they have once melted out, which they will do with or without the presence of micro-organisms, I fear not for any putrefactive micro-organisms which can enter. They are not large enough for you to reach with a probe. They are very minute, and it is impossible for any material, the granules of molecules of which will enter into them, to do harm. Hence the septic condition of the dentinal tubuli does not worry me in the least. If I can but seal the mouths of those tubuli that is all I care for, because the amount of material at that time remaining within the tubuli is so minute that it cannot do harm if the mouths of the tubuli are sealed. There is not sufficient pressure for it to pass through, and not sufficient opening, and the chance or possibility of its passing through the interglobular spaces is so remote that it is a factor that need not be taken into consideration. Having once removed thoroughly the contents of the pulp-canal, having thoroughly sterilized it, having introduced some material that shall seal the mouths of the tubuli, I take my chances on the rest of it. It seems to me it is going into speculative philosophy a little too deep. We all want to be educated to our first best. We have enough men who are given to the second best. We must mark the limits of practicability, however, in our practice and in our reading and in our teaching, and I think we are going too far. We are neglecting more important things when we descend into the minutiæ of this to such a great extent. There is a middle course between what is called the want of scientific treatment, as recommended by Professor Abbott, and the exceedingly minute attention which is given to the subject in the paper. I am quite willing to admit that the safe side is that to which the most attention is given, and yet life is short. There are too many other things for us to give our attention to than to bestow it on these minute tubuli when the source lies in the pulp-canal. We must secure sterility from the ingress of micro-organisms into the pulp-canal.

I think every man should do the best he can. I believe every man is so constituted that he will do the best he can. I believe honesty is the normal condition of mankind, and until we have been badly instructed, until our teaching has been evil, until by precept or example we have adopted bad habits, we will remain honest, and choose what is the best, if we act from the normal impulses which move ordinary human beings. I believe the best sense of the profession is in the line of that which is safest, of the medium course, neither eschewing, upon the one hand, the totally unscientific, and, upon the other hand, this fine splitting of hairs, which is incomprehensible to the average dentist, and yet, as one of those who desire the highest, I can most heartily commend this paper. It is exactly in the line of my own ideas, and that which I most desire to study. I think we are frittering away our energies when we spend them altogether upon the consideration of the things which are more impracticable in practice than they are applicable to the every-day work of the ordinary dentist.

*Dr. M. L. Rhein*, of New York.—While I am in hearty sympathy with the object and tenor of the paper, and it represents to us the present scientific aspect in reference to the septic conditions that we meet in root canals, yet as the author of the paper has seen fit to speak of a paper of mine in reference to this subject, which I read last December before the Second District Society, of Brooklyn, I feel it incumbent upon myself to take issue with him in reference to the statement he makes in regard to the effect of what he calls the coagulating material that I there recommended for washing out the canals after they have been thoroughly cleansed. In this question of the use of coagulants or non-coagulants, I coincide very heartily with the remarks of Dr. Wassall; while theoretically the use of non-coagulants seems to be the proper thing, yet taking this subject into active practice and knowing clinically the effects which we there meet, we are confronted by facts which show us that it is one of those theories which is very much overdrawn, and the actual facts do not follow out the strict line of theory. In other words, the much mooted assertion that a coagulant is a barrier to the further coagulation of the tissue beyond, has been disproved so effectually and so frequently that to my mind it has ceased to exist as a fact. At the same time, I am ready to admit that it is a barrier to the diffusibility of antiseptic remedies that we wish to diffuse through the tubuli; but the author makes a mistake when he says that a solution of bichloride of mercury dissolved in peroxide of hydrogen, which has a certain amount of muriatic or sulphuric acid in it, is a coagulant. The amount of acidity in the peroxide of hydrogen at once nullifies the coagulating effect of the bichloride of mercury, and this fact seems to escape the attention of the author. He has made a decided objection in his paper to what is known as the Schrier method,—

the mixture of potassium and sodium. I agree heartily with his remarks wherein he objects to the treatment, as outlined by Schrier, and that is, merely inoculating the putrid pulp with this material, and not removing it; but I have found no method so effective for removing every portion of pulp-tissue, to the ends of the minutest root, and for penetrating some distance into the tubuli of the periphery of the root-canal, as this mixture of potassium and sodium. There is no reason why we should avoid a good thing because the originator of it has gone a certain distance and stopped there. The only thing necessary to do after thoroughly inoculating the putrid mass with this admirable mixture of potash and sodium is to thoroughly remove the material that has been left behind. It is put in such a condition that it becomes very easy to do. The powerful effect of the potash and sodium leaves the periphery of the canal in such a state that the tubuli are to a great extent more open than with any method of cleansing the root-canals that I have yet been made familiar with, and the syringing out of that canal after the contents have been removed, with a solution of bichloride of mercury in a solution of peroxide of hydrogen, which, as I said, is not a coagulating fluid, causes the penetration of the tubuli to a very great extent of a most powerful germicidal remedy. If the condition of that root, and I quote as nearly as possible from the paper the author speaks of, has been so putrescent or there has been so much trouble beyond the end of the root as to warrant it, if a dressing of a diffusible oil like oil of cinnamon is left in that root-canal for a few days it will diffuse through the tubuli in such a root in a much more rapid manner than I have seen diffusion by any other means of root treatment that I know. I recall a case of that kind in my own family, where I received marked evidence of the cinnamon oil penetrating through the cementum of the root; the taste of it in the mouth was evident forty-eight hours after the cinnamon oil had been placed in the root-canal. The point I wished to make was that this treatment which Dr. Wassall claims is a coagulating treatment and prevents the diffusion, on the contrary, increases the rapidity of the diffusion of an essential oil through the dentinal tubuli.

*Dr. Wassall.*—Dr. Abbott seemed to think that no method could be entirely perfect in preventing a recurrence of trouble. The method that is the most perfect is the one which we should adopt, and we should study the question thoroughly and decide for ourselves which is the best, if we cannot get an absolutely perfect one. He claims he gets perfect antisepsis of the contents of the tubules by the use of sublimate. I merely wish to call his attention to the fact that sublimate is precipitated in the presence of albumen, and is therefore inert. Dr. Rhein does get the effect he desires from using sublimate and peroxide, but the result probably depends on the peroxide alone.

In regard to the point made that immediate root-filling would bring forth a bad odor, I would say that while Dr. Abbott does not have that result in the cases he treats, it is due to his peculiar skill. I have removed many such fillings, and detected a bad odor. If they had been treated for a long enough period of time with diffusive disinfectants, this condition would have been overcome.

Dr. Abbott thinks that the putrefactive matter which occupies the tubules is a substance which may be ignored; that the anatomical difficulties which the interglobular space affords to the passage of putrid matter from the tubules to the cementum is sufficient. I am convinced by observation that in many teeth where the roots have been filled perfectly, that there is soreness of the tooth in the socket, and I am convinced that this is because the putrid matter in the tubules, which we are told is exceedingly poisonous when it decomposes to the extent of forming ptomaines, is the cause of that irritation. Dr. Rhein himself tells us how easy it is for the oil of cinnamon to be detected through the dentine, through the cementum, and through the gum, and be tasted in the mouth, and how easy it is for this poisonous matter to also go through the tooth.

*Dr. Morgan.*—Upon the assumption that the soft tissues in the tooth may be so thoroughly brought under the influence of antiseptics as never to take on putrefactive decomposition again, upon what ground do you insist upon the removal of even the pulp of the tooth after it is sterilized?

*Dr. Wassall.*—I do not believe that the tissues which remain in the dentine become mummified. I am speaking of the putrid state of the organic matter in the tubules. I do not think those materials should stay in the tubules, but ought to be removed. They are removed by a constant application of dressings, by a constant use for twelve or fifteen days of a process of osmosis. Four days after you take out a dressing that has been put in, you find it discolored. It is not matter that was in the pulp-canal, but it is matter that has by osmosis come from the tubules, and under the law of osmosis the drug which was on the dressings would pass into the tubules. The tubules are occupied by the antiseptic dressing after this time, and the putrefied material has entirely disappeared.

Section passed.

The chair appointed as a committee to inquire into the condition of the Dental Protective Association, Drs. Patterson, Hulbert, Perry, Morrison, and Richards.

Section VI., Physiology and Etiology, was then called, and was responded to by Dr. Patterson, chairman. Section passed.

Dr. Brophy stated that Section VII., Anatomy, Pathology, and Surgery, has not yet had a meeting, on account of the meeting of the Association of Faculties. Three papers are to be presented, the first of which is by Dr. W. C. Barrett, of Buffalo, N. Y., entitled "A Study of the Orbicularis-Oris Muscle."

Dr. Fillebrown then read a paper entitled "The Relation of the Frontal Sinus to the Antrum," and reported five cases in his practice, in which the trouble in the antrum could be traced to the frontal sinus.

DISCUSSION.

*Dr. Hunt.*—I only desire to corroborate some of the statements of Dr. Barrett in regard to his paper. The paper related more especially to the function of the orbicularis-oris muscle, as well as giving the minute anatomy of its structure and relation to other muscles of the face. I doubt whether we think enough about the function of this muscle. It acts differently in the upper lip from what it does in the lower lip. For instance, in speaking, the upper lip does not move very much,—hardly at all. It is only in extreme cases, or in certain individuals that the function of the orbicularis is sufficient to cause any great amount of motion in the action of the upper lip, while in the lower lip the function is increased. To make that clear to you, you will find that you can push the lower lip up beyond and over the upper lip; but you cannot push the upper lip over the lower one. As Dr. Barrett said, the fibres are not continuous fibres running directly around the cavity. If no great number of these fibres surrounded the cavity, this function would not be divided in the manner it is. Another operation of the function of the muscle is that in lifting the upper lip you cannot do it without at the same time distending it. The region of the levator muscles does not have very much action. It is very nearly in a fixed position. As Dr. Barrett has finally settled his conclusions on the conditions to be reproduced in an artificial denture, I may be allowed to speak of it, although it is not in the section of anatomy. You cannot move one muscle of the face without moving all of them. No one can act independently. They can be made to do so by an unusual education or training of some sets of muscles, like the contortionist employs. Indications of this are found in the habit that is retained in the use of the lip by individuals long after the teeth are lost, the habit being contracted in early life from an abnormal condition of the orbicularis-oris caused by an abnormal condition of one or two of the teeth. For instance, if, in the process of the incoming of the teeth, the lateral incisor and bicuspid come so closely in contact that the cuspid does not get its normal condition, standing out in such a way that the individual contracts a habit of holding the lip in that way, that habit will continue. The cuspid tooth has a fixed position. In the chart showing the levating and nasal muscles the proprius muscle should be set a little farther back from the angulus oris. Consistency in the position of the muscles is not the rule. Variation is a common condition, and we have a variation in the position of the muscles very often in the same mouth. In studying the effect of the functions of these muscles we must study them all together for the purpose of education. You can hardly do so differently than Dr. Barrett did in the paper, taking up the function of each muscle individually, and yet they all act together. This is the point I desire to impress particularly,—that in the study of the question you must take into consideration all the muscles of the face. Whether they are in their normal position or not will have much to do with the final result. A general statement, perhaps, that will be of more value is that in the pleasing motions all the lines of the face as produced by the muscles are radiating from the central portion of the face upward and outward. In all of the disagreeable expressions these lines are downward and inward to the central portion of the face. That is the only general idea or thought that underlies the use of the function of these muscles for a final result.

I desire to speak as to the effect of the restoration of these muscles to an analogous position that they occupy normally in the mouth. In an aged person, where resorption has occurred, it is not possible always to restore these features without the extraordinary use of material. Dr. Eames, three or four years ago, made a suggestion which I think, if you will utilize it, will be of considerable value. In extreme resorption it is not alone the alveolar border that resorbs, but the muscles themselves droop. (Illustrating.) He suggested not only raising this point as high or a little higher than you ordinarily get it with an impression, but at the same time building over a hood, so to speak, not making a mass of material to correspond with this projection, simply making an inflection over the margin of the plate. You will find the effect is simply this,—it does not restore the features outwardly, but it lifts the orbicularis-oris muscle. In doing that it naturally controls all the muscles that act with the orbicularis-oris muscle. Many of the difficulties that we have been compelled to meet will be eradicated by that method. It can even be continued in extreme cases back to the region of the last molar tooth, which will add in some cases to the efficacy of the result.

*Dr. Corydon-Palmer.*—The subject of adaptation to the face is one that has been my study from the time of Dr. John Allen, in 1842, when I met him in Cincinnati, all the way through his experiments, and adding to them my own, and adding his criticism on what I was doing. I am able to show you now the result. You must allow me to go back a little into history to show the things we had to use. In 1843 the artificial teeth were not furnished to us as they are now; they were in mass, and a little later they were placed in boxes, like tacks. There were no full sets. At my suggestion Mr. White put the teeth on cards, so when we wanted an incisor that was the right size we could see at once. There were no first or second bicuspids in New York in 1843, and no finishing molar. At my suggestion, the teeth were arranged on cards from the furnishing of my models. There were first and second bicuspids, right and left, and a finishing molar which had never been in existence, because in making my pieces I placed on my plate a little metal that represented what should be a finishing molar. Teeth were of different lengths and did not match. At my suggestion, the teeth were made in entire sets, and my models were afterwards copied. We then had furnished to us molars that were long enough for the restoration of the features. (Illustrating.) Supposing this represents the left superior side of the mouth. This line would lift up the muscles and bring out the features. In an ordinary piece this would go straight across. It has been my aim to bring this point forward and have it well understood. In reference to Dr. Eames's idea of raising the muscle on each side of the nose and getting a different expression in that way, his position was false. When Dr. John Allen showed me his piece, which was remarkable because he was the first one who made what was considered restoration by filling up the mouth, he did not endeavor to do more than put in a quantity of something that would fill up the mouth, but in my studies I endeavored to bring back exactly the lost portion and represent it as perfectly as I could; have it prominent where it was wanted, have it sunken where it was wanted, and so restore the shape of what was lost by extraction or absorption. Dr. Allen said to me at Clinton Falls, where we held our meeting many years ago, when I took him aside and exhibited to him my piece, that it was all right, but I should not leave any hole for the cavity in the extension. It is unnatural and will cause a whistling sound. As is often said, nature abhors a vacuum. It must be rounded and smooth, and there must be nothing there to interfere with the articulation and the sound. So Dr. Eames was wrong in carrying up his work and folding it over. I have not put in a book what I have done, but the older men know what I have done, and I want to tell the younger men that I have given my vital forces and life energy in this work. I have never asked for any money or any design or model I have furnished. I have given everything to the associations and to the profession. I have something more that I wish to bring forward, and I will do it at your pleasure. The most important fact I wanted to bring forth is, let it conform as nearly to the original shape and form as possible. Try to exactly reproduce the lost part. Professor Barrett surprised me by making his report. I want to show you that it is true. Here is a piece which I have in my own mouth. It was made without having a brush put upon it. I trimmed it up and burnished it myself.

*Dr. Barrett.*—It pleased me especially because Dr. Palmer said he had never studied the names of many of these muscles, and he did not know much about it; but he has made allowance for every muscle which I have shown on my charts here to-day.

The chairman announced that the organization of sections would now take place.

Adjournment.

(To be continued.)

